# Developing a Healthy Web-Based Cookbook for Pediatric Cancer Patients and Survivors: Rationale and Methods

**DOI:** 10.2196/resprot.3777

**Published:** 2015-03-31

**Authors:** Rhea Li, Margaret Raber, Joya Chandra

**Affiliations:** ^1^MD Anderson Children’s Cancer HospitalDepartment of Pediatrics-ResearchThe University of Texas MD Anderson Cancer CenterHouston, TXUnited States

**Keywords:** obesity, pediatric cancer, survivorship, nutrition, cooking

## Abstract

**Background:**

Obesity has been a growing problem among children and adolescents in the United States for a number of decades. Childhood cancer survivors (CCS) are more susceptible to the downstream health consequences of obesity such as cardiovascular disease, endocrine issues, and risk of cancer recurrence due to late effects of treatment and suboptimal dietary and physical activity habits.

**Objective:**

The objective of this study was to document the development of a Web-based cookbook of healthy recipes and nutrition resources to help enable pediatric cancer patients and survivors to lead healthier lifestyles.

**Methods:**

The Web-based cookbook, named “@TheTable”, was created by a committee of researchers, a registered dietitian, patients and family members, a hospital chef, and community advisors and donors. Recipes were collected from several sources including recipe contests and social media. We incorporated advice from current patients, parents, and CCS.

**Results:**

Over 400 recipes, searchable by several categories and with accompanying nutritional information, are currently available on the website. In addition to healthy recipes, social media functionality and cooking videos are integrated into the website. The website also features nutrition information resources including nutrition and cooking tip sheets available on several subjects.

**Conclusions:**

The “@TheTable” website is a unique resource for promoting healthy lifestyles spanning pediatric oncology prevention, treatment, and survivorship. Through evaluations of the website’s current and future use, as well as incorporation into interventions designed to promote energy balance, we will continue to adapt and build this unique resource to serve cancer patients, survivors, and the general public.

## Introduction

### Childhood Cancer Survivors and Obesity

Obesity has been a growing problem among children and adolescents in the United States for a number of decades. According to the Centers for Disease Control and Prevention, in 2012, nearly 20% of children ages 6-11 were obese, compared to only 7% in 1980; among adolescents (ages 12-19), 21% were obese, compared to 5% in 1980 [1]. This alarming trend is mirrored in survivors of childhood cancers, which is a population that has benefited from improved treatments, leading to a cure rate of 83% [2]. However, childhood cancer survivors (CCS) are more susceptible to the downstream health consequences of obesity such as cardiovascular disease, endocrine issues, and risk of cancer recurrence due to treatment received [3-5]. Therefore, obesity prevention in this population requires specific action.

Unhealthy eating habits developed during treatment routinely persist into survivorship. For example, chemotherapy often changes the taste and smell of foods, decreases appetite, increases satiety, and causes nausea and vomiting [6,7]. Frequently, parents and providers are permissive of allowing patients to succumb to poor food choices to accommodate these issues. As a result, strategies to promote healthy diets in CCS must include the continuum of cancer care, beginning with treatment. A cross-sectional survey conducted to determine CCS quality of life found that most survivors did not meet recommended dietary and physical activity guidelines [8]. CCS also reported an interest in computer-based healthy lifestyle interventions [8]. Another survey found that most parents and CCS desired to live a healthier lifestyle, but their behavior fell short [9]. CCS may also adopt sedentary behaviors and poor eating habits, which contribute to chronic health issues [5,10].

### “@TheTable”

In an effort to build resources that enable pediatric cancer patients and survivors to lead healthier lifestyles, we developed a Web-based cookbook called “@TheTable”. Freely available, this website provides unique features that address healthy eating during treatment and throughout survivorship. The website is mobile phone, with Internet access capability, and tablet compatible and recipes are searchable by many criteria including symptoms. Also, individual recipe ingredients can be added and subtracted in real time, with caloric information adjusting immediately. Here, we describe the development process, novel aspects, and current and future applications for the cookbook website.

## Methods

### Creating the Website

The “@TheTable” website was created by a committee of individuals associated with the University of Texas MD Anderson (MDACC) including a registered dietitian, patients and family members, a hospital chef, and community advisors and donors. Research staff organized the sourcing and testing of recipes and managed the entry of recipes and caloric information into Web-based databases. An in-house core facility at MDACC, called e-Health Technology, was commissioned to build the website with the information provided by research staff.

### Collecting the Recipes

**Figure 1:**
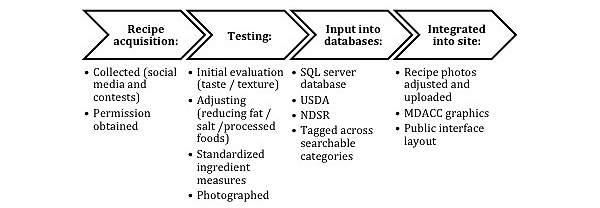
Recipe inclusion process: scheme depicting how recipes were collected, tested, and uploaded into the "@TheTable" database. Abbreviations: SQL:Structured Query Language; NDSR: Nutrient Database; MDACC: MD Anderson Cancer Center; USDA: United States Department of Agriculture.

Figure 1 shows the process of adding recipes to the cookbook. Recipes were collected from MDACC patients, employees, local restaurants, chefs, the Houston Culinary Guild, and through contests on social media, including the University of Texas MD Anderson Children’s Cancer Hospital’s (MDA-CCH) Facebook and Twitter pages. The committee set guidelines to ensure recipes were simple and nutritious including a 10-15 ingredient limit per recipe for 4-6 servings, usage of common household ingredients, 10 or fewer simple steps, a suggested total preparation time of 60 minutes or less, and a recommendation of fewer than 400 calories per serving. After recipes were received, they were tested and evaluated by research staff and volunteers. Evaluation forms were created to ensure quality control during recipe testing. Recipes were standardized to US measures (cups, tbsp, etc) and changes were made to enhance recipe quality, taste, and/or nutrition. These adjustments were communicated to the recipe author before being added to the cookbook. Final versions of the recipes were tasted and evaluated by staff; patients also evaluated select dishes. Each recipe was photographed. Research staff wrote instructions and descriptions, and each recipe was marked for inclusion in various search categories. The MDACC graphics department and the MDACC core facility, e-Health Technology, created the layout and presentation of the public interface, allowing users to search for, view, and alter recipes, as well as access other media.

Data entry of recipes and caloric information was facilitated by the e-Health Technology group. A recipe database was provided to researchers, which calculated nutrition information using the US Department of Agriculture National Nutrient Database for Standard Reference [11] for macronutrient content. Many recipes were also analyzed using Nutrition Data System for Research software (2013) developed by the Nutrition Coordinating Center, University of Minnesota, Minneapolis, MN [12] for micronutrient composition.

The e-Health Technology back-end system included a structured query language (SQL) server database, interactive user interface design, and a secure network for data collection/transfer. The SQL server database was designed to maintain user information for researchers, recipe details, searchable recipe tags, user ratings, user comments, and resource links. Researchers with administrative access to the database had the ability to input, change and alter recipe information, add new searchable tags, upload videos and pictures, and upload tip sheets. The public user interface of the system allows for users to navigate the recipe bank with searchable tags, interactively customize recipe ingredients, rate recipes, comment on recipes, share recipes through social media, and access all other media content including tip sheets and videos. The website was designed to be fully functional with tablets and mobile phones with Internet access capability.

In order to provide a resource that was both useful and unique to patients and survivors, we sought out the advice and personal experiences of the MDA-CCH Family Advisory and Adolescent/Young Adult Advisory Council members, which included current patients, parents, and CCS. Feedback included adding nutrition analysis for each recipe, an interactive search feature, colorful designs, and the ability to alter ingredients and have the changes reflected in the nutrition facts label. The council members also provided valuable feedback regarding search categories that may be important to CCS and patients including color, texture, taste, and specific nutrients.

Nutrition tip sheets were developed based on advice from council members and staff from several disease-specific sections within the MDA-CCH. Videos demonstrating recipes were created by research staff in collaboration with University of Texas-Television and uploaded to the website, as well as to YouTube. The video “chefs” include research staff, volunteers, and patients. These videos and tip sheets are publically available on the Internet.

### Presenting the Cookbook

A media tour was planned and executed to disseminate the cookbook including 11 radio interviews and meetings with reporters at three top-tier parenting publications. Further, research staff went to each MDA-CCH disease-specific section to present the cookbook and gather feedback for its improvement. The cookbook was also presented at several conferences and promoted at hospital events.

To determine if similar resources existed and to allow “@TheTable” to be unique in its format, other Web-based cookbooks were researched. Websites such as “Eat to Beat”; “Cook for Your Life”; and the National Heart, Lung, and Blood Institute’s (NHLBI) healthy eating cookbook all provide healthy recipes, cooking tips, and cooking demonstration videos [13-15]. The “Eat to Beat” website focuses on the prevention of cancer, while the “Cook for Your Life” website provides recipes for the cancer patient [13,15]. The NHLBI Web-based cookbook provides heart healthy recipes for the family [14]. However, none of these cookbooks offer healthy recipes tailored specifically to the pediatric cancer patient or survivor and their families. They do not have as many search options inclusive of symptoms, food textures, and meal preparation time, nor do they offer real time nutrition content adjustment of recipes.

## Results

### “@TheTable” Website Recipes

**Figure 2:**
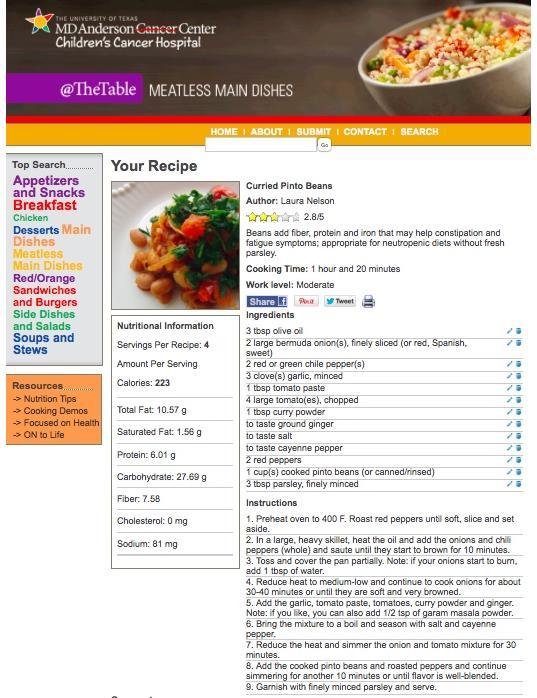
Screenshot of a recipe from "@TheTable" cookbook website.

Initial planning and decision making for the cookbook began in summer 2010. The first hundred recipes were tested and entered into the database by midsummer 2012. The “@TheTable” website officially launched in the fall of 2012. To date, over 400 recipes have been indexed on the “@TheTable” website, which is freely available on the Internet [16]. A registered dietitian at MDA-CCH has analyzed all included recipes. Nutritional information is available for each recipe and automatically adjusts when users change ingredient amounts (Figure 2 shows this).

Specifically, the ingredients list can be manipulated by altering ingredient amounts (double, half, or quarter recipe) or by deleting ingredients completely. For example, by clicking on the blue pencil icon illustrated in Figure 2, ingredient amounts can be doubled, halved, or quartered. The blue trash bin icon in Figure 2 will delete the ingredient completely. Ingredient alterations immediately change the accompanying nutrition information panel. This allows users to observe how certain ingredients contribute to a recipe’s nutrition profile. Recipes are tagged across several categories so they are searchable by type of dish, ingredient, color, nutrition, cooking time, taste, and texture. Descriptions and examples of search terms are available in Table 1. All search terms were chosen based on feedback from MDA-CCH patient and family advisory councils.

**Table 1:** Detailed search terms and descriptions available for the “@TheTable” cookbook website.

Search category title	Description/reasoning	Available search terms
Type of dish	Describes meal courses and specific foods	Breakfast, appetizers/snacks, soups/stews, sandwiches/burgers, main dishes, meatless main dishes, side dishes/salads, desserts, dips/sauces/gravies, beverages/smoothies
Color	Overall color of the dish; feedback provided that children may prefer foods of certain colors	Red/orange, green, blue/purple, white/yellow
Nutrition related recipes	Terms that define the health of the dish; patients requested finding dishes that tailored to a specific nutrient need	Low sodium low fat, fiber, iron, protein, vitamin K, low cholesterol, zinc, calcium, vitamin C, magnesium
Quick meals	Total amount of time to complete a recipe	4 time ranges, under 15 minutes, 15-30 minutes, 30-45 minutes, 45-60 minutes
Symptom related recipes	Recipes that contain foods with potential for alleviating side effects	Neutropenia, diarrhea, constipation, weight gain, nausea/vomiting, dry mouth/mouth sores, fatigue, platelets
Taste	Patients reported preferences for certain sensory foods during treatment	Salty/savory, sweet, sour/tangy, spicy, strong flavor/aroma
Texture	Children have preferences toward foods of a specific consistency	Crunchy, smooth/creamy, soft, dry, chewy

### Cooking Videos

In addition to healthy recipes, social media functionality and cooking videos are also built into the website. Specifically, users are able to rate and comment on each recipe or share their favorite recipe on Facebook, Twitter, or Pinterest. Cooking videos featuring local chefs, staff, and pediatric cancer patients/survivors demonstrating recipes found in the cookbook were filmed onsite at MDA-CCH.

The “@TheTable” website is also a nutrition information resource. The website has 26 nutrition and cooking tip sheets available with topics ranging from cooking, shopping, and food storage suggestions to more general nutritional information. The website is continuously updated with new recipes, videos, and nutrition information and maintained by research staff in collaboration with the MDA-CCH site managers.

## Discussion

### “@TheTable” as a Resource for Interventions

With over 400 recipes in the database, and ongoing efforts to include more, “@TheTable” is a unique resource for promoting healthy lifestyles spanning pediatric oncology prevention, treatment, and survivorship. Several research projects in various stages of completion are utilizing the Web-based cookbook. During the cookbook’s infancy stage, a personalized monthly weight management counseling intervention for pediatric cancer patients was developed. This intervention utilized recipes from the cookbook to provide healthy cooking tips and nutrition advice to patients and their families, who reported the website was helpful for finding recipes tailored to a specific symptom.

The “@TheTable” website is also being used as a cancer prevention resource for patients, survivors, and the general public. Recipes from the website are used for employee wellness programs and monthly cooking classes for optimal health. While currently just a resource and not an evaluation tool, the Web-based cookbook could be incorporated into assessment-oriented interventions. This is a major emphasis of future studies by our group. For example, a community weight management program for overweight children was designed using an entire curriculum of recipes from “@TheTable”, as a cancer prevention intervention. Participants were encouraged to visit the website to find recipes that were demonstrated during each weekly cooking class. Feedback from this program is currently being analyzed.

### Future Goals

In addition, video game-based behavioral interventions that integrate the cookbook with gameplay are underway. Another future goal is to increase accessibility of this resource to Hispanic populations. Recipes and tip sheets in Spanish are currently limited in availability on the website; however, there are plans to incorporate more Spanish recipes, videos, and resources.

Through evaluations of the website’s current and future use, as well as incorporation of the website into interventions designed to promote energy balance, we will adapt and build this resource to serve cancer patients, survivors, and the general public.

## Abbreviations

CCS: childhood cancer survivors
MDACC: University of Texas MD Anderson
MDA-CCH: University of Texas MD Anderson Children’s Cancer Hospital
NHLBI: National Heart, Lung, and Blood Institute
SQL: structured query language
